# 111 years of allergen-immunotherapy: A long and successful history of the only available disease-modifier in allergic diseases 

**DOI:** 10.5414/ALX02330E

**Published:** 2022-11-21

**Authors:** Jan Gutermuth, Martine Grosber, Oliver Pfaar, Karl Christian Bergmann, Johannes Ring

**Affiliations:** 1 Department of Dermatology, University Hospital Free University Brussels, Brussels, Belgium,; 2 Department of Otorhinolaryngology, Head and Neck Surgery, Section of Rhinology and Allergy, University Hospital Marburg, Philipps-Universität Marburg, Marburg,; 3 Institute of Allergology, Charité – Universitätsmedizin Berlin, Corporate Member of Freie Universität Berlin and Humboldt-Universität zu Berlin, Berlin, and; 4 Department of Dermatology and Allergology Biederstein, Technical University Munich, Munich, Germany

**Keywords:** adjuvants, allergoids, allergen immunotherapy, hyposensitization, history, oral immunotherapy in food allergy, sublingual immunotherapy

## Abstract

The great milestones in medicine almost always have their precursors, which help the great event to break through. So it was with allergen-specific immunotherapy (AIT) and the great work of Noon and Freeman and their world-renowned publication in 1911. In this article, we want to outline AIT’s long journey, from early attempts to achieve tolerance to allergens in the environment. Many very different methods were used; from homeopathy to the use of recombinant allergens. Initially, the allergen extracts were given only subcutaneously, but then also through other routes, such as nasal, rectal, intradermal, epicutaneous, in lymph nodes, or oral. It was the great merit of Bill Franklin, whom many of us still experienced as active participants in congresses, to point out that the effect of AIT must be documented not only by clinical observation but in a controlled form including placebo injections. AIT was thus transferred to evidence-based medicine, which we successfully apply today. We would like to express our gratitude to Bill Franklin himself and all others involved in the development of AIT with this summary of 111 years of immunotherapy.

## Introduction 

Allergen immunotherapy (AIT) (also called specific immunotherapy (SIT), allergen-specific immunotherapy (ASIT), desensitization, hyposensitization) is the administration of a specifically relevant allergen for the treatment of IgE-mediated allergic diseases [1, 2, 3, 4, 5, 6] through the sublingual or subcutaneous route. 

Most publications see the introduction of this only available disease-modifying treatment option for allergic patients in 1911, referring to an article by Leonard Noon in the Lancet [7]. However, the idea was not totally new, there were some “precursors”. 

## Early precursors 

The principle of inducing clinical tolerance against a harmful stimulus has been tried already 2,000 years ago by king Mithradates from Pontos who wanted to protect himself against being poisoned. King Mithradates VI. (132 – 63 B.C.) took increasing doses of snake venom to make himself immune against the venom. It is unknown whether this treatment really worked, but there is some probability, because when the Romans defeated him, he had to kill himself with a sword [8]. Scientifically – if it worked at all – it could be explained by a phenomenon like tachyphylaxis. 

A more realistic precursor of AIT was the development of vaccination for protecting humans against smallpox infection using putrid secretions from cowpox. The observation was coming to Europe by the British Lady Montague in Turkey and then used by Edward Jenner (Figure 1). who developed a true “vaccine” and gave the name since the material was from cows [9]. He could prove the effects and introduced large vaccination programs, first in England and later all over Europe. It is interesting to note that the first country in the world where a smallpox vaccination was obligatory by law was the Kingdom of Bavaria in 1807 [10]. 

At the same time, Samuel Hahnemann brought forward the concept of “homeopathy” based on the observation that the same substances that can induce harm sometimes also may be able to induce protection or cure [11]. According to the philosophy “similia similibus” he tried to treat several diseases with dilutions of the respective elicitors [11]. Hahnemann himself was a scientist, contrary to his successors who continued to use his ideas almost like a religion and exaggerated the phenomenon of dilution by calling it “potentiation”. The biggest difference between homeopathy and AIT is that AIT follows a dose-response regimen, and its effects have been proven by many randomized double-blind placebo controlled clinical trials [12]. 

## Early AIT attempt around the turn of the 19^th^/20^th^ century 

**Figure 2:**
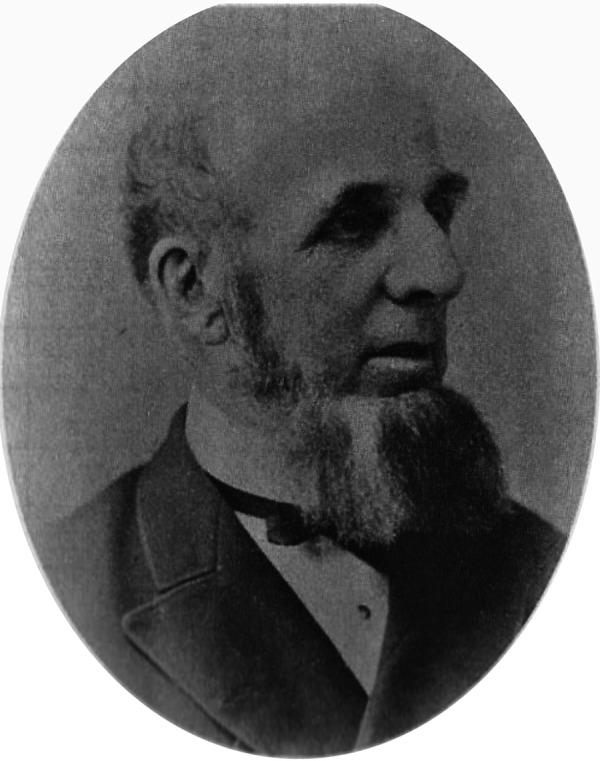
Charles Blackley.

The breakthrough of scientific allergology came at the end of the 19^th^ century following the seminal work of Charles Blackley [13] who proved that pollen are the cause of hay fever and that he could measure the sensitization by skin and provocation tests (Blackley 1873 [13], Figure 2). 

**Figure 3:**
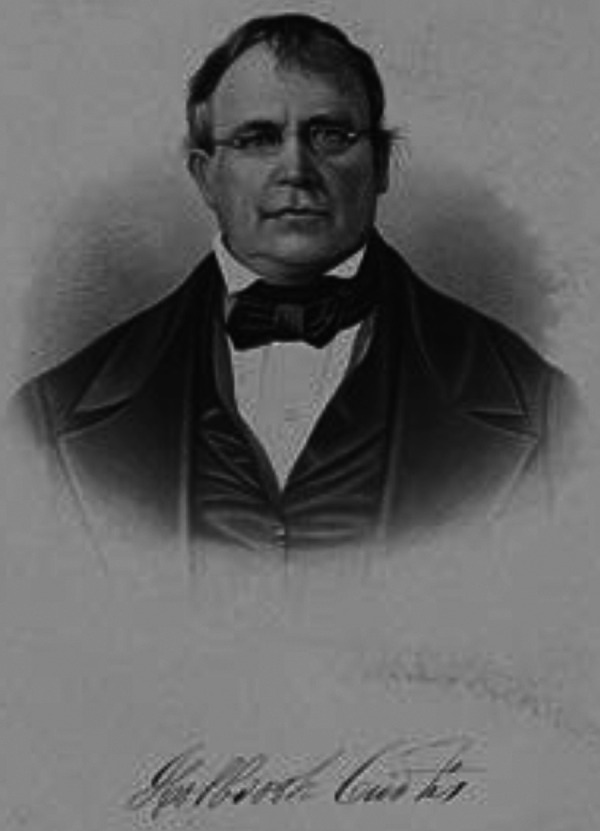
H. Holbrooke Curtis.

Already in 1899, the laryngologist H. Holbrock Curtis [14] (Figure 3) published an exciting article on “The immunizing cure of hay fever” in the Medical News of New York 1900 . In one case, he used subcutaneous injections of an extract of flowers and pollen. Later he treated a small series of patients with a pollen extract, probably by oral application. 

Also in the United States of America, Rosenau and Anderson [15] observed that the application of heterologous sera (e.g., horse) induced immunity in individuals previously reacting with anaphylaxis. Clemens von Pirquet [16] (Figure 4) had observed similar findings which he noted in his book “Serum sickness” describing a self-experiment on doctor a certain “Doctor v.P.” who showed decreasing local reactions of the skin after intradermal injections. 

As early as 1903, Philip Dunbar [17] in Hamburg tested the concept of protective immunity which had been developed in the treatment of diphtheria using anti-toxins by Behring [18] who received the first noble prize in Physiology or Medicine in 1901. He produced an hyperimmune serum in animals immunized with pollen extracts, called “pollantin” [17]. Probably this procedure was not really successful due to anaphylactic reactions against the animal serum. 

At the same time, Alfred Wolff-Eisner, an immunologist and allergist in Berlin, realized that proteins in the pollen are the triggers of hay fever and performed a pilot study in hay fever patients using the ophthalmo test [19]. This study was not really taken up by the scientific community since it was published in German language in the Berlin “Monatsblätter für Augenheilkunde” [20]. Thus, one cannot criticize Leonard Noon for not quoting his colleague from Germany. 

**Figure 5:**
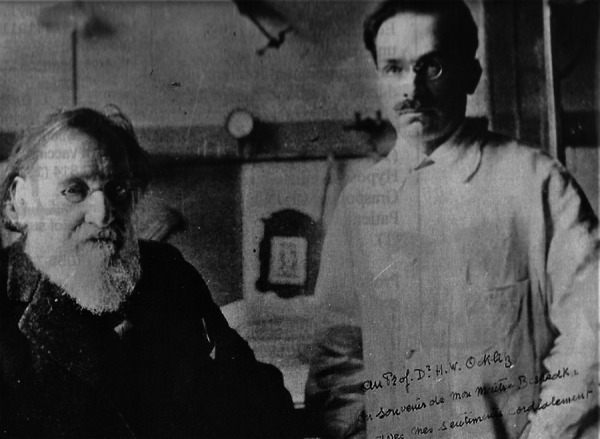
Alexandre Besredka (1870 – 1940) at the right, next to Alexandre Elias Metchnikoff at the Institute Pasteur, Paris.

In France, Alexandre Besredka [21] (Figure 5), a student of Metchnikoff, who believed that anaphylaxis also occurred in the brain – an early precursor of the concept of “psycho-neuro-immunology” – performed animal experiments with “vaccination anti-anaphylactique” with increasing doses of diluted allergen solutions. 

In a kind of local immunotherapy, Scheppegrell [22] used a powder of dried pollen applied into the nose as nasal therapy in patients with hay fever. 

## Noon’s and Freeman’s work 

In 1911 Leonard Noon [7] published his seminal article on “prophylactic inocculation against hay fever” in the Lancet (Figure 6). He believed that hay fever is caused by a “soluble toxin” in pollen which is “innocuous to normal individuals”. To detect this “idiosyncrasy” he used an ophthalmo test, which is what we call today a conjunctival provocation test. After studying dose response curves in order to find the right amount, he treated patients with subcutaneous injections of aqueous extracts of pollen from different grasses and could show a reduction of the conjunctival and nasal reactions after the treatment and also provided future prospects to investigate “whether the immunity thus attained is sufficient to carry the patients through a season”, evidence filled by many clinical trials in the 20^th^ century [12, 23, 24]. 

Unfortunately, Leonard Noon died too young from tuberculosis in 1912, but his work was continued by his colleague John Freeman who published “Further observations on the treatment of hay fever by hypodermic inoculations of pollen vaccine” ([25] (Figure 7). After these articles had appeared, the procedure was taken up rapidly all over the world by physicians treating hay fever patients. Yet it took decades until the first double-blind, placebo-controlled controlled trial was performed. In the 1950s, William “Bill” Frankland in England, who had worked with John Freeman and also with Alexander Fleming, performed a first controlled trial proving that hyposensitization was significantly more effective in higher doses than in a lower dose or when using placebo in the treatment of hay fever [26]. Bill Frankland was an enthusiastic and dedicated physician and researcher who inspired many young allergists not only in the United Kingdom (Figure 8). After a rich and adventurous life – he served as an army physician in Singapore and became imprisoned by the Japanese on the so-called “Hell’s Island” – he died of COVID-19 in 2020 at the age of 108 years [27, 28]. 

## Application routes of AIT 

AIT is used in many modalities with regard to extract production, modification, adjuvants used, and route of application. In addition to subcutaneous application, other routes have been tried, like oral, nasal, rectal, intradermal, or epicutaneous. 

Also injection into a local lymph node was tested in order to induce a stronger immune response [29, 30]. 

**Figure 9:**
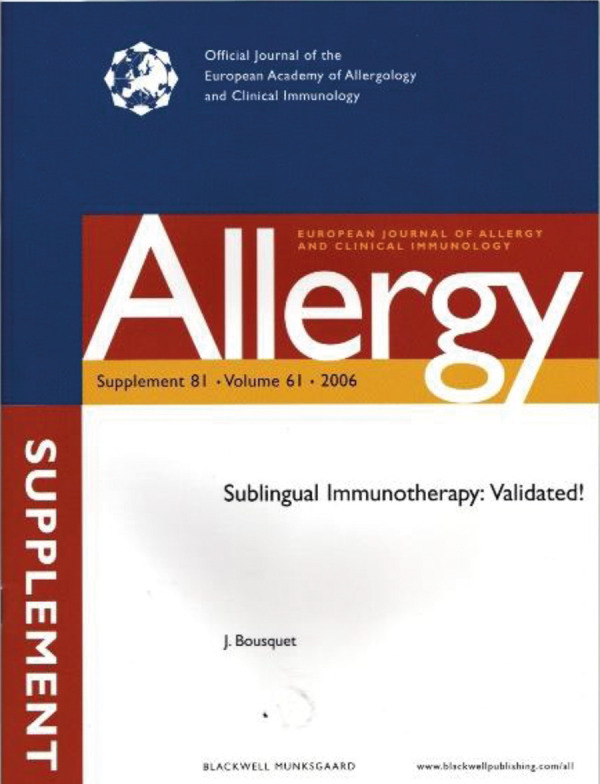
Cover page of the journal “Allergy”.

At the end of the last millennium there was a heated and long-lasting debate between supporters of subcutaneous immunotherapy (SCIT) against the newly coming up oral [31, 32, 33, 34] or sublingual application (SLIT) [35]. This debate is closed (Figure 9): sublingual immunotherapy is as effective as the subcutaneous one, which has been proven by a number of excellent and large randomized placebo-controlled clinical trials [36, 37, 38]. Sublingual immunotherapy uses aqueous solutions as droplets and, more recently, lyophilized allergens tablets. 

## Innovations in SCIT as the first described AIT application route 

Usually, aqueous extracts that were applied through the subcutaneous route (subcutaneous immunotherapy (SCIT)) were used for AIT. 

A major problem was the purification of allergens from the plants or animals or house dust. Then, with improved purification procedures, standardization was crucial. In order to compare different allergen extracts, a variety of arbitrary units were used starting with the classical weight/volume (WV) units according to Noon, then advancing to the protein content as protein nitrogen units (PNU), or also describing biological activity with histamine equivalent prick (HEP) or biological allergy units (BAU). 

Furthermore, attempts to modify the allergens by chemical treatment were undertaken with the concept of “allergoids”; modification was done by formaldehyde or glutaraldehyde leading to preparations with increased immunogenicity but reduced allergenicity, meaning the frequency of adverse side reactions [39, 40]. 

Also a variety of adjuvants were used together with the allergens in order to increase the efficacy of the immunological effect in analogy to classical vaccines. Therefore, the most commonly used adjuvant was aluminum hydroxide in the form of semi-depot allergen preparations. Other modalities include tyrosine, calcium phosphate, and monophosphoryl lipid (MPL) [41]. Also in scientific studies, polyethylene glycol-coupled allergens were used [42], and later the Th1-stimulating CpG oligonucleotides [43]. 

## Insect venom allergy 

**Figure 10:**
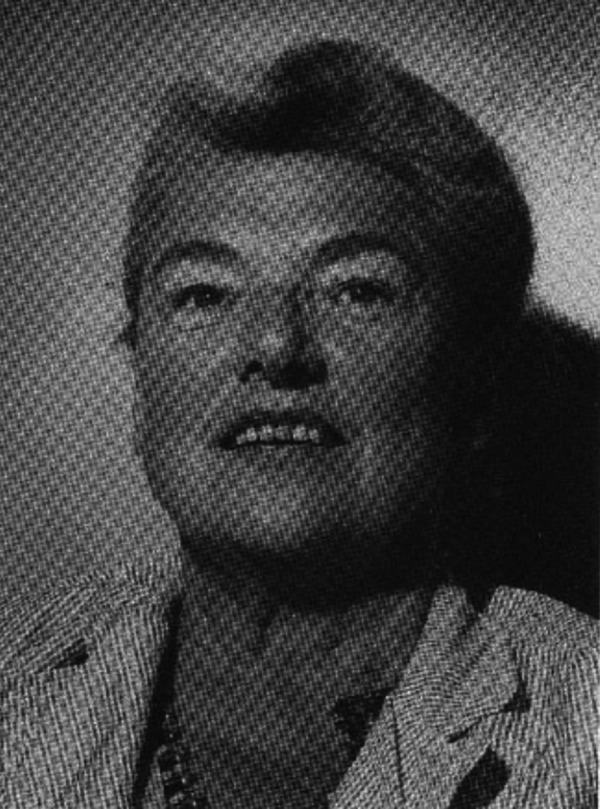
Mary Hewitt Loveless.

A special chapter in the history of allergen-specific immunotherapy is the treatment of insect venom anaphylaxis. This starts with the observation that beekeepers rarely develop anaphylaxis, while certain allergic individuals with insect venom allergy can suffer from fatal reactions. Therefore, hyposensitization was also tried to treat these conditions. Based on a note by Benson and Semenov who treated a beekeeper, who was anaphylactic and also suffered from asthma, with whole-body extract from bees. They found improvement [44]. Based on this report, a decades-long tradition of using whole-body extracts from bee and wasps started and even was accepted by official documents of the American Academy of Allergy. At the same time, Doctor Mary Loveless (Figure 10) was able to transfer the immunity with beekeeper’s serum thus establishing the concept of “blocking” antibodies [45]. The hobby beekeeper and gifted pharmacist Heinrich Mack from Illertissen, Southern Germany, had the idea to use electrical stimulation to produce a purified bee venom solution which he developed for treating rheumatic diseases under the name “Forapin” [46]. This Forapin was also used by allergists in the 1930s for hyposensitization in bee-sting-induced anaphylaxis [47]. Probably due to side effects, this treatment has never becoming routine. 

It took until the 1970s in the United States when Larry Liechtenstein and co-workers in Baltimore performed the first double-blind placebo-controlled randomized clinical trial in insect venom allergy using placebo, whole-body extract, and purified bee venom. Whole-body extract was not better than placebo with still 40% of improvement, while bee venom showed an efficacy of over 90% [48]. This study also already showed the often high placebo response rate making clinical studies in allergology so difficult. 

## Recombinant allergens 

A vision of modern allergy researchers was to overcome the standardization problems of allergen extracts by using recombinant allergens, since the molecular nature of major allergens had become known [49]. Some studies indeed showed efficacy of recombinant allergens both from bee venom [50] and from grass pollen [51]. Yet, due to technical and economic as well as regulatory aspects, no company finally was able to transform this concept into routine procedures. So allergists are still dreaming of measuring the allergenic potency of extracts in nanograms or micrograms of relevant molecularly defined allergens. 

## Guidelines 

At the end of the 20^th^ century, national or regional guidelines for allergen specific immunotherapy were developed in many countries and regions and also at an international level under the auspices of the World Allergy Organization [52]. Throughout the recent two decades, an increasing number of national and international systematic reviews, position papers, and guidelines have been published in the field of AIT (some examples: [3, 53, 54, 55]). 

The guidelines are following internationally recognized standards, such as the AGREE-II or GRADE methodology, in consenting clinical recommendations based on solid evidence [12, 23, 24]. In addition several aspects of improving methodological standards in conducting clinical trials and assessing efficacy of AIT have been elaborated by international panels of experts in the field [4, 56, 57, 58, 59]. 

Special emphasis is given to the evidence available for various allergen preparations used in the treatment of allergic diseases. There is no generic efficacy in AIT (in both application routes SCIT and SLIT), and a product-specific evaluation of the safety and efficacy of the individual product investigated in clinical trials is mandatory. 

## Food allergy 

While AIT is obviously effective in airway allergy, there is still a need for patients suffering from food allergy, especially food anaphylaxis [60]. While single case reports and pilot studies have shown efficacy of oral immunotherapy in food allergy, this has not become routine [61]. However, early desensitization protocols have been described already 50 years ago and have been further improved for patients with cow’s milk allergy by Wüthrich [62] in the subsequent decades. 

Recently, a standardized preparation has been registered for treating peanut allergy by oral immunotherapy [63]. Other products for subcutaneous use are in the pipeline. 

## Atopic eczema 

While atopic eczema/dermatitis used to be a contraindication for AIT, this is no longer the case. However, the indication for AIT to treat this skin disease is still not supported by the majority of guidelines, although there are pilot studies and also placebo-controlled randomized trials showing a beneficial effect in atopic dermatitis/eczema [64]. 

## AIT during the COVID-19 pandemic 

While COVID-19 emerged as a global pandemic as declared by the WHO in March 2020, it also impacted all medical disciplines. Very early during the pandemic, international guidance has been given by the European Academy of Allergy and Clinical Immunology (EAACI) aimed to ensure that patients with respiratory allergic diseases are adequately treated under pandemic restrictions [65, 66, 67, 68]. In addition, straightforward and consensus-based recommendations for both SCIT- and SLIT-treated patients have been proposed internationally [69] and have also been adapted to the situation in German-speaking countries [70]. However, a high grade of heterogeneity of adherence to the academic guidance given has been found in international [71] and national [72] surveys on COVID-19 and AIT suggesting some undertreatment of patients. 

## Future concepts 

Over the last 20 years, many exciting and new therapeutic concepts have been followed in animal experimental and clinical studies, for instance coupling of allergens with microbial products, virus-like particles, or combination with biologics dampening the Th2 response; also T-cell-based epitope vaccines have been developed but have not reached routine clinical use [5, 73]. 

Also hybrid molecules [74] have been used equally as hypoallergenic iso-allergens [75]. DNA vaccination and gene therapy have been discussed and studied in experimental models [76, 77]. 

An original idea came from Switzerland with a concept of immunizing cats to induce neutralizing antibodies against the major allergen Fel d 1 [78]. 

## Conclusion 

The last 111 years – or 120 if we consider the early scientific reports – can be regarded as a success story of immunologic treatment of allergic diseases. AIT is the only causal treatment of a variety of common allergic diseases besides allergen avoidance, which is not entirely possible for most allergies. With AIT it is possible to modify the pathogenic immune deviation towards tolerance after contact with allergens. Together with allergy diagnosis it remains the backbone of allergological experience which was and is the individual (“personalized”) management of patients suffering from allergic diseases. 

## Funding 

None. 

## Conflict of interest 

JG, OP, KCB and JR declare no conflict of interest. 

**Figure 7:**
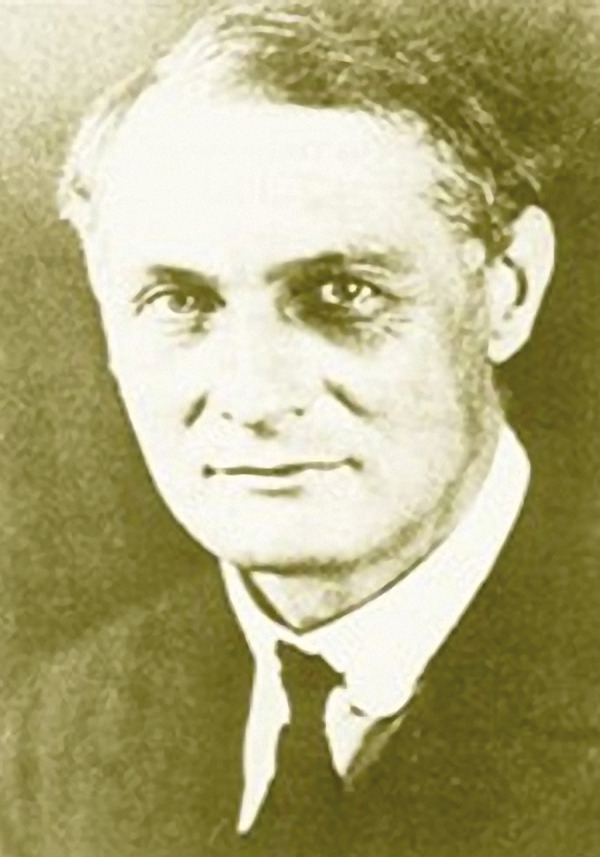
John Freeman (1877 – 1962).

**Figure 8:**
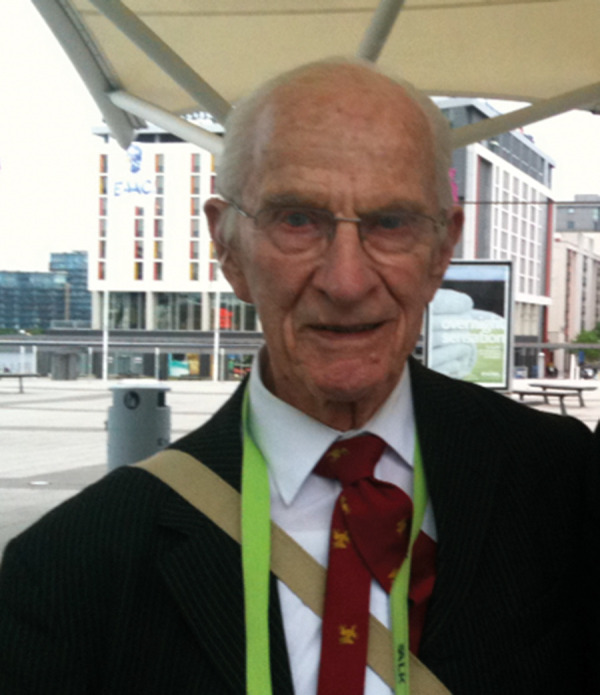
Alfred William „Bill“ Frankland (1912 – 2020) one of the top allergists of the 20^th^ century. Foto: Oliver Pfaar.

**Figure 6:**
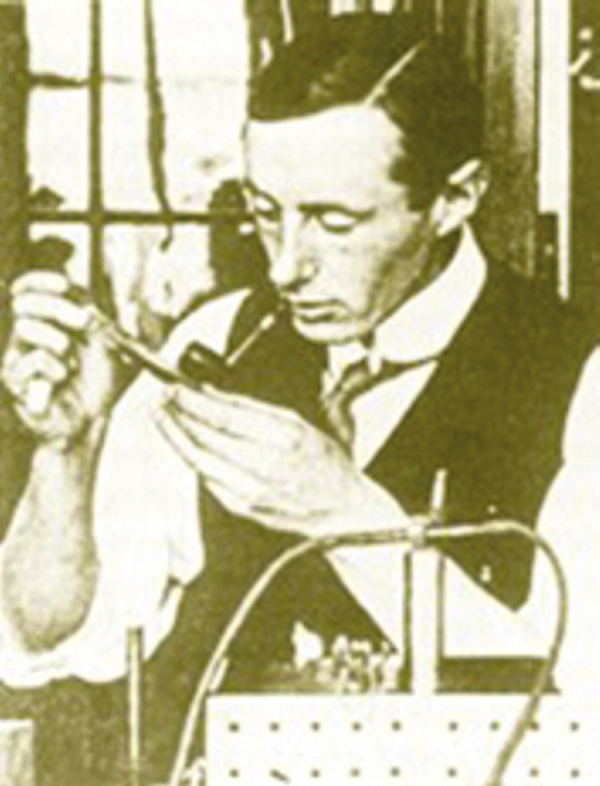
Leonard Noon (1878 – 1913) in the laboratory.

**Figure 4:**
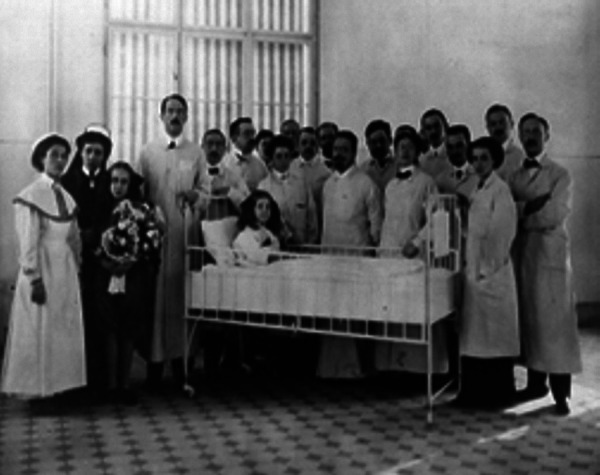
Clemens Peter Freiherr von Pirquet ( 1874 – 1929) surrounded by colleagues and nurses.

**Figure 1:**
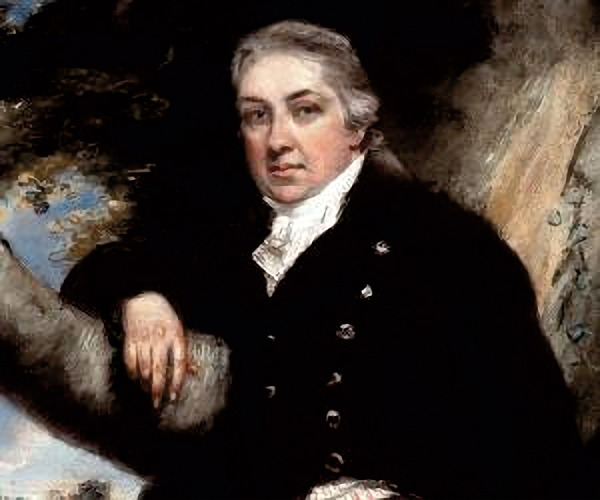
Edward Jenner (1749 – 1823) English doctor who helped create and popularize a vaccination for smallpox.
